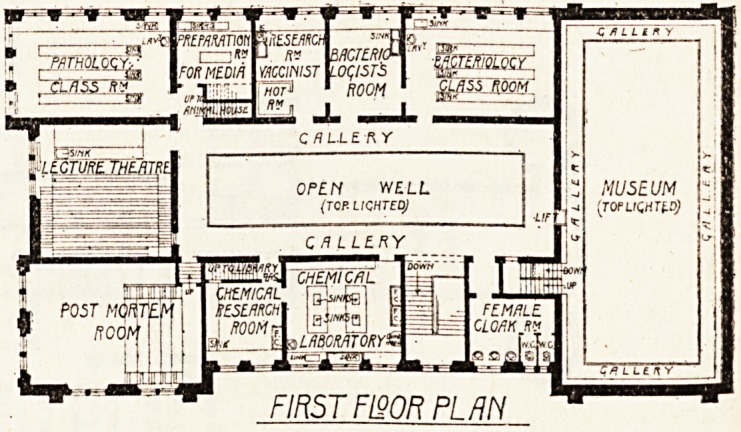# The New Pathological Building at Glasgow Royal Infirmary

**Published:** 1914-11-28

**Authors:** 


					The New Pathological Building at Glasgow Royal Infirmary.
The pathological building has been planned by
Mr. James Miller, F.R.I.B.A., the architect to the
infirmary, with the collaboration and assistance of
the trustees, managers, and medical staff. There is
a central hall of fine proportions with circular roof
and top lights. A gallery runs all round this hall
which gives access to the various laboratories and
lecture rooms. A notable feature is the light-
ing, which has been most excellently planned and
arranged. The post-mortem room also has a gal-
lery for students and is well ventilated. In con-
nection with it cold storage and refrigerating
apparatus have been placed in the basement, which
also contains the mortuary and has a small chapel
adjacent to it. The building contains good class
rooms, a lecture theatre, a chemical laboratory
with a research room attached, and good accommo*
dation for the bacteriologist, the pathologist, and for
research purposes. The central hall has made it-
possible for the architect to arrange each depart-
ment with due regard to the efficient working and
teaching arrangements of the pathological building-
PATHOLOGICAL BUILDING ?
/o 2o y> to so -to . refi
4-
mi
\mxmm wit
Ljtt
\=^\OfFICi
_ ?
11
?//77?/7A'C?
JAMES MILLER fKXMCtf
6R0UND F150R FLfiN '^?%v00?
j/TESE/mm
1bacterid
pfiTHNCCY - I L- . r n- VHULMV-
M FOR MEDIA \MCC1N15T l.0(;i5T5 . ^
l j r:; | HOfl 1 room 1 g^JSSQM^
By
'k&mTJWmi
JL
^ | _ CHL.LERY
It
|
i post mokt
" ffOOAj
?LJ
OPEN WELL I !* MUSEUM
(TOP-LIGHTED) I (TWLMip;
QALLERY
&& E
[aj'"?{nj |
LmmoRYft
3CSK-:
tof/fsmj-
TOOM'H
?3:
FEMALE
CLOAK JW
5ES' "* ?
FIRST FI90R PL AN

				

## Figures and Tables

**Figure f1:**
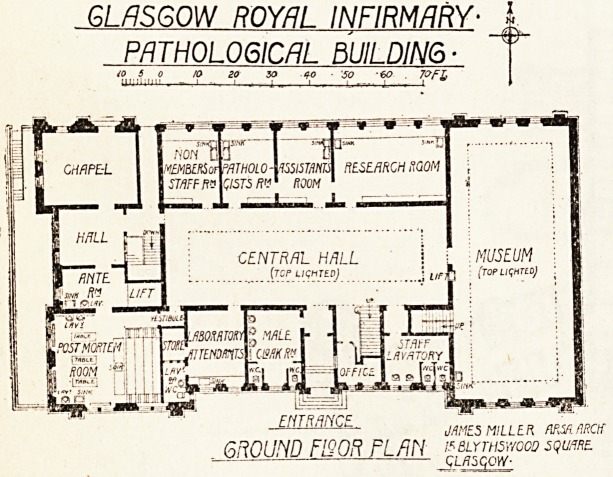


**Figure f2:**